# Effect of pharmacokinetically-relevant ivermectin concentrations on survivorship and fecundity of *Anopheles coluzzii* and *Aedes aegypti* in Burkina Faso: A laboratory experimental study

**DOI:** 10.1371/journal.pone.0332677

**Published:** 2026-03-27

**Authors:** Emmanuel Sougué, Cheick Oumar W. Ouédraogo, Fabrice A. Somé, Greg Pugh, André B. Sagna, S. Rodrigue Dah, Saoudatou Bagayogo, Risnahar T. Ouédraogo, Mady Ndiaye, Sunil Parikh, El Hadji M. Niang, Brian D. Foy, Roch K. Dabiré

**Affiliations:** 1 Institut de Recherche en Sciences de la Santé, Direction Régionale de l’Ouest, Bobo-Dioulasso, Burkina Faso; 2 Université Cheikh Anta Diop de Dakar, Sénégal; 3 Center for Vector-borne Infectious Diseases, Department of Microbiology, Immunology and Pathology, Colorado State University, Fort Collins, Colorado, United States of America; 4 ASTRE, CIRAD-INRAE, Univ Montpellier, Centre de Recherche et de Veille sur les Maladies Vectorielles dans la Caraïbe (CRVC), Petit Bourg, Guadeloupe, France; 5 Department of Epidemiology of Microbial Diseases, Yale School of Public Health, New Haven, Connecticut, United States of America; Vector Control Research Centre, INDIA

## Abstract

**Background:**

The control of vector-borne diseases is increasingly challenged by insecticide resistance widespread. Therefore, innovative vector control tools with alternative modes of action are urgently needed to support existing ones. Ivermectin (IVM), a broad-spectrum endectocide has been shown to exert lethal effects on mosquitoes, including *Anopheles* and *Aedes* species following blood meals taken from treated humans or livestock. In this study, we assessed the effect of IVM at concentrations equivalent to human plasma levels following mass drug administration (MDA), on the survival and fecundity of *An. coluzzii* and *Ae. aegypti* in Burkina Faso. The present study provides new insights into how IVM may impact differentially both malaria and arbovirus vectors, contributing to develop integrated strategies of vectors control.

**Methods:**

Two laboratory experiments were conducted using 3–5-days old wild-derived female *An. coluzzii* and *Ae. aegypti*. Each experiment included four replicates per IVM concentration and was performed on separate dates. Mosquitoes were membrane fed with rabbit blood treated with five IVM concentrations (C = 112 ng/mL, C2 = 29 ng/mL, C3 = 15 ng/mL, C4 = 6.5 ng/mL, C5 = 2.5ng/mL), corresponding to the mean human plasma levels at 2, 4, 7, 14, and 28 days post-MDA with IVM at a dose of 3x300 µg/kg. A negative control with free-IVM (0.0 ng/mL) was also included. Mosquito mortalities were recorded daily for 7 days. Fecundity was measured by counting both laid and developed eggs (via ovary dissection).

**Results:**

IVM significantly reduced the survival of *An. coluzzii* compared to the control group (*p < 0.001*), with the risk of death increasing from 4.2-fold at the lowest concentration (2.5 ng/mL) to 64.2-fold at the highest (112 ng/mL). In contrast, IVM had no significant effect on *Ae. aegypti* (*p > 0.05*), and the survival rate of control group did not differ from that of treatment groups. Additionally, for *An. coluzzii*, IVM significantly reduced both egg laying and egg development (p < 0.0001 and p < 0.001, respectively), whereas no significant impact on fecundity was observed in *Ae. aegypti* (all *p > 0.80)*.

**Conclusion:**

IVM concentrations typically achieved in human plasma during MDA campaigns were sufficient to significantly reduce both survival and fecundity of wild type *An. coluzzii*, but had no measurable effect on *Ae. aegypti*. These findings highlight the species-specific response to IVM and support its potential role in integrated vector control strategies targeting malaria vectors in Africa.

## Introduction

Vector-borne diseases such as malaria and dengue remain major public health challenges, affecting millions of people worldwide each year. In 2023, the World Health Organization (WHO) estimated approximately 263 million malaria cases and approximately 597,000 related deaths with about 95% occurring in African countries, where many at-risk populations still lack access to preventive measures [1]. In Burkina Faso, approximately 8,139,000 malaria cases and 16,146 deaths were recorded in the same year [1]. Dengue fever also surged in 2023, with five million cases and over 5,000 deaths worldwide reported by WHO. In Africa, Burkina Faso was the most affected country during this outbreak with 146,878 suspected cases, 68,346 confirmed by Rapid Diagnostic Test (RDT) and 688 associated deaths [2]. These data highlight the co-endemicity of malaria and dengue in Burkina Faso.

In areas where malaria and dengue are co-endemic, vector control strategies primarily rely on indoor residual spraying (IRS), long-lasting insecticide-treated bed nets (LLINs) and larval source management (LSM) [3]. These interventions use mainly pyrethroids insecticides, that target the voltage-gated sodium channels in mosquito neurons. However, the effectiveness of these tools is increasingly compromised by insecticide resistance widespread [4] and mosquitoes behavioral changes including shifts in biting times (earlier or later), biting and resting outside of human dwellings (exophagy and exophily), and feeding preference (zoophagy) [5–7]. This growing challenge of multi-factorial resistance and the continued propagation of vector borne diseases around the world [8] highlight the urgent need for novel vector control tools that can target mosquitoes regardless of their biting or/and resting behavior, [9].

IVM, a semi-synthetic derivative of avermectin is mainly used to treat parasitic diseases such as onchocerciasis, lymphatic filariasis headlice, strongyloidiasis and scabies [10,11]and was approved for human use since 1987 [12]. It is well tolerated in both humans and animals and widely used in MDA campaigns for controlling parasitic infections [13]. Beyond its anti-parasitic properties, IVM has demonstrated mosquitocidal effect in several studies [14,15], leading to its consideration as a novel tool for vector control [16,17]. Indeed, unlike conventional public health insecticides, IVM targets the glutamate-gated chloride channels in mosquito nerve and muscle cells, causing an uncontrolled influx of chloride ions, resulting in paralysis and death [18,19]. The recommended human dose during MDA is 150–200 µg/kg body weight, typically resulting in peak plasma concentrations of 40–45 ng/mL [20]. These levels have been shown to reduce survival rates of *Anopheles* mosquitoes for 5–7 days post-treatment [21,22]. Due to its safety profile, frequently administration of high IVM doses can increase and maintain plasmatic concentrations for a long time [23]. In our study, the corresponding plasma concentrations used were derived from pharmacokinetic measurements in participants treated with IVM at a dose of 300 µg/kg x 3 day-course, estimated by height bands.

Although several studies have assessed the effects of IVM on the survival of *Anopheles* and *Aedes* mosquitoes, the majority have relied on laboratory-colonized strains. Consequently, its impact on wild-type *Anopheles* and *Aedes* remain insufficiently explored, despite the fact that these vectors are often sympatric and frequently feed on the same human hosts [24]. A laboratory experiment was performed to investigate the effects of IVM on survival and fecundity of wild-type *An. coluzzii* and *Ae. aegypti*, which are sympatric and the primary vectors of malaria and dengue in Burkina Faso.

## Materials and methods

### Mosquito collection and rearing

Gravid *An. coluzzii* females were collected from human dwellings in Bama (11°23’14”N,4°24’42”W) in January and May 2024. The first sampling was conducted on January 26, 2024 for blood fed experiment round 1 and the second sampling took place on May 2, 2024 for the experiment round 2. The collected mosquitoes were placed individually in cups containing water and covered with mesh netting to allow oviposition. Species were confirmed by routine PCR [25] after oviposition. All *An. coluzzii* larvae were pooled and reared in tap water and fed with Tetramin® Baby Fish food (Tetrawerke, Melle, Germany).

*Ae. aegypti* used was a wild-derived type, originating from larvae collected in breeding sites around the city of Bobo-Dioulasso (11°10’37”N, 4°14’52”W) in October 2022. The colonies were repeatedly refreshed with larvae collection on the field.

All mosquitoes were under insectary standard conditions (temperature: 27 ± 2°C; relative humidity: 70 ± 10%; photoperiod: 12h light followed by 12h dark).

### IVM preparation

**Fig 1:**
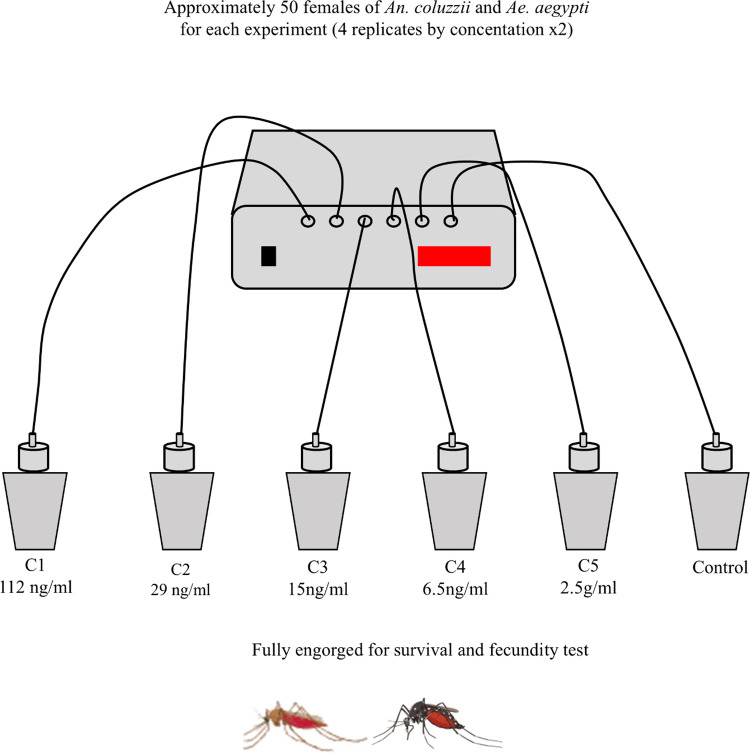
Experimental workflow.

Powdered IVM was obtained from Sigma-Aldrich (St. Louis, MO, USA). A stock solution was prepared at a concentration of 10^4^ ng/mL after the dilution of IVM powder in dimethyl sulfoxide (DMSO) and refrigerated overnight at 4°C. This stock solution was then diluted in DMSO to create three working solutions of 10^3^ ng/mL, 10^2^ ng/mL and 10 ng/mL (**S1 Fig** **1**). From the 10 ng/mL solution, five concentrations (C1 = 112 ng/mL, C2 = 29 ng/mL, C3 = 15 ng/mL, C4 = 6.5 ng/mL, and C5 = 2.5 ng/mL) corresponding to mean IVM plasma concentrations measured at days 2, 4, 7, 14, and 28 days, respectively, following MDA with IVM were prepared. The values were based on pharmacokinetic/pharmacodynamic (PK/PD) data from a sub-study of the RIMDAMAL II trial [26]. Each concentration was mixed with rabbit blood for use in mosquito bioassays. The adequate volume of rabbit blood and of the IVM solution at concentration of 10 ng/mL was mixed, giving the final working solutions for a final volume of 1 mL (blood + IVM). Adequate volume of rabbit blood and of IVM solution to be pipetted was calculated using the formula.

S1 Fig: IVM dilution and concentration preparation.

### Feeding bioassays

Blood-feeding assays were conducted using freshly collected rabbit blood on each experimental day. For both mosquito species, two independent experiments were conducted on different dates, with four replicates per IVM concentration. For *An. coluzzii*, the first experiment was performed on February 10, and the second on May 17, 2024. The first experiment with *Ae. aegypti,* was performed on March 4, and the second on April 8, 2024. For *An. coluzzii*, the survival tests were carried out in 4 replicates for each experiment and across all concentrations (C1, C2, C3, C4, C5, and control). In contrast, *Ae. Aegypti* was evaluated only at concentrations C1, C2, and the control using the same number of replicates and experiments like *An. coluzzii*. Across both strain in each replicate, four test cups containing approximately 50 females aged 3–5 days-old were used. Females were starved of sugar for 8 hours prior to feeding assays. Blood meals were offered using Hemotek membrane feeders (Hemotek Ltd., UK), covered with parafilm heated at 37°C, and placed on top of each cup. Mosquitoes were exposed to IVM-treated blood for 30 minutes (**Fig 1**). After feeding, unfed and partially blood fed females were discarded and only fully-engorged were maintained in the insectary at 27–29°C, ~ 70% relative humidity (RH) with access to water and 10% sucrose for 7 days. The study protocol has been approved by the institutional ethical committee with the CERS reference number 2019-01-009.

### Survival and fecundity assessment

Mosquito survival was monitored daily, and dead mosquitoes were removed. Mosquitoes were considered dead if unable to stand on their legs.

For fecundity assessments, approximately 50 unfed females were fed with blood containing different IVM concentrations (four replicates per concentration). Fecundity assays started 3 days post exposure to IVM, due to high mortality during the first two days after feeding. Only surviving mosquitoes were kept in labelled cups, where petri dishes containing moist cotton and filter paper were provided as oviposition support for gravid females. Mosquitoes were maintained for five days, with access to 10% sucrose solution. Eggs laid on filter paper were counted under binocular microscope at 2X magnification. Afterward, all mosquitoes were dissected to examine their ovaries and count retained eggs that were not laid.

### Statistical analysis

Data were entered and cleaned using Microsoft Excel 2021, and statistical analyses were performed using R software version 4.4.1. Mosquito survival data for each species, were visualized using Kaplan-Meier curves, and differences among IVM concentrations were assessed using the Log-rank Mantel-Cox test and the Kruskal-Wallis non-parametric ANOVA was used as a post-hoc test. In this analysis the number of mosquitoes survived days post-treatment was considered as response variable for each concentration. Hazards ratios (HRs) and their 95% confidence intervals were estimated using time-to-event analyses, employing Cox proportional hazards models to evaluate associations between variables. Lethal concentrations (LC values) were estimated using non-linear regression analyses with the *drc* package for R software [27]. Generalized linear regression models were employed to assess the impact of IVM on egg production for surviving female mosquitoes. The mean (95% confidence interval) number of developed and/or laid eggs per female according IVM concentration were calculated. All *p* values <0.05 were considered statistically significant and retained in the minimal adequate model [28].

## Results

### Effect of IVM on mosquito survival

A total of 2,400 *An. coluzzii* and 1,200 *Ae. aegypti* females were used to assess the effect of IVM on mosquito survival. After exposure to IVM-treated blood, 1,290 *An. coluzzii* and 548 *Ae. aegypti* were fully engorged and used for survival monitoring.

Results indicated that IVM concentration had no significant effect on blood-feeding rates in both species. *An. coluzzii* blood-feeding rates were 47%, 51.5%, 53.5%, 52.2%, 52.7%, and 67% for concentration C1 (112 ng/mL), C2 (29 ng/mL), C3 (15 ng/mL), C4 (6.5 ng/mL), C5 (2.5 ng/mL), and control, respectively. Blood-feeding rates of *Ae. aegypti* were 39.25%, 46.5%, and 51.25 for C1 (112ng/mL), C2 (29ng/mL) and control, respectively.

**Fig 2:**
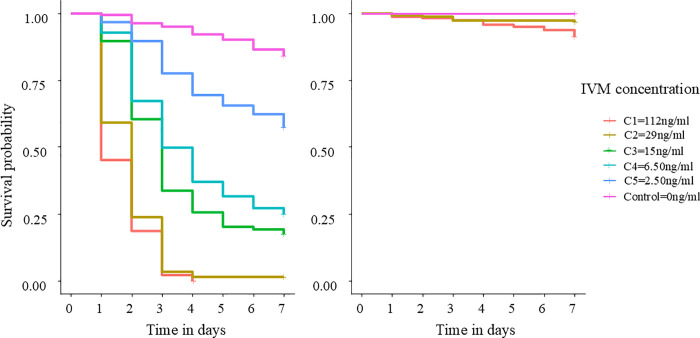
Kaplan-Meier curve showing the survival probability of *An. coluzzii* (left) and *Ae. aegypti* (right) over post-treatment according to IVM concentration.

Survival probabilities of both species over 7 days are shown in **Fig 2**. IVM had no significant effect on the survival of *Ae. aegypti* (*p > 0.05*). On day 7 post-ingestion, survival remained at 100% in the control group and approximately 95% for the two concentrations tested (C1 and C2; **Fig 2**). As survival in *Ae. aegypti* stayed almost the same even with highest IVM concentrations, further tests with lowest concentration were stopped, LCs values and Hazard Ratios (HRs) could not be estimated for this species.

In contrast, *An. coluzzii* showed high survival in the control group, while survival was significantly reduced in all IVM-treated groups (Likelihood Ratio Test, LRT χ^2^ = 960.9, df = 5, *p <* 0.001, **Fig 2**). At day 1 after blood feeding, survival was reduced by 50% with C1 (112ng/mL), 35% with C2 (29 ng/mL), 13% with C3 (15 ng/mL), 7% with C4 (6.50 ng/mL) and 3% with C5 (2.50 ng/mL). By day 3, survival dropped further to reach approximately 5% for both C1 (112ng/mL) and C2 (29ng/mL), 35% for C3 (15 ng/mL), 50% for C4 (6.50 ng/mL), and 76.56% for C5 (2.50 ng/mL). Survival fell to 0% for C1 (112 ng/mL) by day 4, while remaining stable at approximately 5% until day 7 post-feeding for C2 (29 ng/mL). *An. coluzzii* survival continued to drop until day 7 post-ingestion for the 3 remaining concentrations to reach 23% for C3 (15 ng/mL), 28% for C4 (6.50 ng/mL), and 65% C5 (2.50 ng/mL).

**Fig 3:**
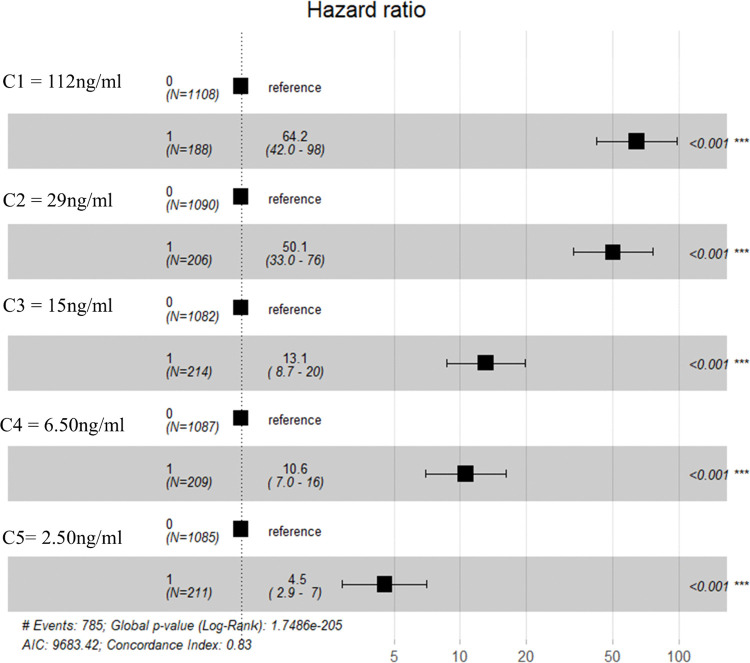
Estimated values of 7-day-post-treatment hazard ratio for *An. coluzzii.*

All IVM concentrations had a statistically significant negative effect on *An. coluzzii* survival when compared individually to the control group. Mosquitoes that fed on IVM-treated blood were more likely to die compared to the control group (**Fig 3**). The 7-days HRs were ≥50 for C1 (112 ng/mL) and C2 (29 ng/mL), between 10 and 20 for C3 (15 ng/mL) and C4 (6.50 ng/mL), and below 10 for C5 (2.50 ng/mL).

Dose-response curves at days 1, 3, and 7 are presented in **S2 Fig**, illustrating cumulative mortality across different IVM concentrations during the 7-day observation period. Estimated lethal concentrations (LCs) resulting in 20%, 50%, and 90% mortality are summarized in Table 1. The LC50 (lethal concentration for 50% mortality) varied depending on the time point used for calculation. At day 1 post-ingestion, the LC50 was around 75 ng/mL. When calculated over three days, it dropped to 9 ng/mL, and further decreased to 7 ng/mL by day 7 (**Table 1**).

**Table 1:** Lethal concentrations (LC) of IVM to *An. coluzzii* calculated at days 1, 3 and 7 post-blood feeding with their 95% CI.

LC (%)	Day 1	Day 3	Day 7
20	20.84 [17.623–24.44]	3.85 [3.11–4.59]	1.92 [1.38–2.45]
50	75.18 [59.80–90.57]	9.44 [8.31–10.56]	7.07 [5.40–8.74]
90	574.16 [312.61–836.61]	39.10 [31.43–46.99]	55.87 [24.18–87.56]

LC = Lethal concentration.

S2 Fig: *An. coluzzii* dose-response curves to IVM at days 1, 3 and 7.

### Effect of IVM on mosquitoes’ fecundity

IVM significantly reduced egg-laying in *An. coluzzii*, but showed no impact in *Ae. aegypti.*

*Fecundity* could not be assessed for *An. coluzzii* at IVM concentrations C1, C2, and C3 due to high post-feeding mortality. Exposure of *An. coluzzii* to IVM (C4 and C5) significantly reduced both the number of eggs laid (F = 32.14, df = 2, p < 0.001) and the number of developed eggs in the abdomen (F = 7.41, df = 2, p < 0.001). The mean number of laid eggs was 11.5 for C4, 57.2 for C5, and 105.1 for the control group. After abdomens dissection, the mean number of developed eggs was 50.6, 54.7, and 62.6 for C4, C5, and the control group, respectively (**Table 2**).

**Table 2:** Fecundity of surviving *An. coluzzii* and *Ae. aegypti* females after treatment with different concentrations of IVM.

Species	Treatment	Number of eggs developed per female mosquito that develop eggs [IC95] (number of females)	Number of eggs laid per female mosquito that laid eggs [IC95] (number of females)
*An. coluzzii*	C4 (6.5 ng/mL)	50.57 [43.4–57.7] (n = 7; N = 68)	11.50 [10.3–23.3] (n = 2; N = 68)
	C5 (2.5 ng/mL)	54.70 [47.1–62.3] (n = 27; N = 80)	57.20 [54.4–60] (n = 27; N = 80)
	Control (0 ng/mL)	62.65 [50.7–74.6] (n = 21; N = 56)	105.10 [89–121] (n = 21; N = 56)
*Ae. aegypti*	C1 (112 ng/mL)	79.50 [66.5–92.5] (n = 14; N = 48)	75.05 [64–86.1] (n = 21; N = 68)
	C2 (29 ng/mL)	63.88 [46.5–81.3] (n = 16; N = 41)	79.95 [65.6–94.3] (n = 18; N = 41)
	Control (0 ng/mL)	66.10 [49.5–82.7] (n = 20; N = 44)	79.85 [66.5–93.2] (n = 20; N = 44)

n = number of females that developed eggs in ovaries or number of females that laid eggs.

N = total number of females for fecundity assays.

For *Ae. aegypti*, fecundity assays were only conducted at C1 and C2 as these high concentrations had shown no significant lethal impact. Unlike *An. coluzzii*, fecundity of *Ae. aegypti* surviving females was not significantly affected by IVM exposure. There were no significant differences in the mean number of eggs laid (F = 32.14, df = 2, p = 0.88) or developed eggs in the abdomen (F = 0.16, df = 2, p = 0.85) between the control and treatment groups. Indeed, the mean number of laid eggs was 75.1; 79.9, and 79.8 for C1, C2, and the control group, respectively. The mean number of developed eggs in the abdomen were 79.5 for C1, 63.9 for C2, and 66.1 for the control group (**Table 2**).

## Discussion

This study evaluated the impact of IVM at concentrations equivalent to human plasma levels following MDA on the survival and fecundity of *An. coluzzii* and *Ae. aegypti* in Burkina Faso. In contrast with many previous studies that used laboratory-susceptible mosquito strains [22,29–35], the present study used derived F1 wild-type and recently colonized mosquito for experiments. Wild-type *An. coluzzii* and recently-colonized *Ae. aegypti* mosquitoes known to be resistant to pyrethroids [36–38] were used. IVM was delivered via membrane feeding using treated rabbit blood, mimicking plasma concentrations observed in human blood at 2, 4, 7, 14, and 28 days post-MDA with IVM at a dose of 3x300µg/kg. Findings demonstrate a clear species-specific difference in IVM susceptibility. All tested concentrations affected the survival and fecundity of *An. coluzzii* females, while no significant effect in both parameters was found for *Ae. aegypti*.

The survival of *An. coluzzii* mosquitoes decreased to 50% at day 1 post IVM ingestion with an LC50 of 75.18 ng/mL. These results are comparable to those reported in a previous study [35]. Another study also revealed that when *An. arabiensis* mosquitoes blood fed on IVM-treated humans, 1–4 days after treatment their survival was significantly reduced [39]. Our study showed that the impact of IVM on mosquitoes is dose- and species-dependent. These results corroborate findings from previous studies demonstrating differential susceptibility of different mosquito species to IVM [22,34]. The survival of wild type *An. coluzzii* mosquitoes in our study decreased significantly when they fed on blood containing IVM and seven-day LC_50_ dose was 7.07ng/mL, in concordance with previous studies where seven-day LC_50_ for *An. gambiae* ranged between 3.35 to 55.60ng/mL [31,32,34,40]. When *An. coluzzii* mosquitoes blood fed on C1(112ng/mL), the majority died within 3 days, and practically all mosquitoes died by day 7 post exposure. The same trend was reported by prior investigation with more than 90% of blood fed females dying by day 6 after ingestion of blood taken from IVM treated patients soon after treatment [29]. When *An. coluzzii* mosquitoes fed on IVM-treated blood groups C2 = 29ng/mL, C3 = 15ng/mL, and C4 = 6.50ng/mL corresponding to the concentrations found on days 4, 7 and 14 post-dosing with 3x300µg/kg, respectively, more than 50% died within 7 days. These results are similar to previous findings in which Foley and *al*. [30] reported that *An. farauti* survival decreased significantly after day 14 when they blood fed on treated humans. Also, a recent field study conducted in Burkina Faso showed that survival rate of mosquitoes collected in villages one week after MDA with IVM decreased comparatively to those collected placebo-treated villages [26]. However, results derived from wild-type mosquitoes that blood fed directly on IVM treated humans and collected directly from the field may be subjected to bias, as these mosquitoes are often relatively old and may die from natural causes rather than as a result of IVM exposure. Additionally, field-collected mosquitoes may contain in their bodies, microorganisms such as parasites, viruses or bacteria like *Wolbachia*, which could affect mosquitoes’ survival [41–43]. In contrast, the F1-generation mosquitoes used were of known age, thus their survival rates can be consequently attributable to IVM. Furthermore, the mortality provoked by the IVM concentration 2.5ng/mL corresponding to the IVM plasma level at day 28 after treatment with high dose remained marginally significant for *An. coluzzii*. Indeed, at this concentration about 40% of blood fed females died. However, Somé and *al.* did not observe a lethal effect of IVM day 28 after MDA in their cluster randomized field trial. This lack of effect may reflect the variability in pharmacokinetic profiles of participants, as well as differences in mosquito collection and testing methods between their field study and controlled laboratory conditions of the present work [26]. Although the lethal effect observed in the field seems to dissipate by day 28 post MDA, IVM remains a complementary tool for vector control as long as the life span of mosquitoes is reduced to prevent them from taking another blood meal and potentially transmit malaria parasites [44].

Contrary to *An. coluzzii*, *Ae. aegypti* displayed a stronger tolerance to all IVM tested concentrations. Survival of *Ae. aegypti* did not decreased significantly after feeding on blood containing IVM at any of the tested concentrations, which reflects data from previous studies suggesting higher IVM concentrations are required to induce lethal and sublethal effect on *Ae. aegypti* [17,45]. This apparent tolerance could be explained by a lower binding affinity of IVM and/or a reduced modulatory effect on glutamate-gated chloride channels in *Ae. aegypti* compared to *An. coluzzii* [22]*.* A previous study suggested that the relative tolerance of some mosquitoes species to IVM could be attributed to at least three reasons: 1) there may be poor absorption of IVM throughout the midgut, 2) there may be physiological differences in glutamate-gated chloride channel structures among mosquitoes species, and 3) some species may be more efficient in their ability to detoxify IVM compared to others [22]. Understanding this tolerance may be particularly critical for further development of IVM-based tools to help control species like *Ae. aegypti* and limit their spread of arboviral diseases.

Fecundity investigation of surviving females after feeding on IVM treated blood revealed a significant reduction of the number of laid and developed eggs compared to the control group, only for *An. coluzzii*. This sub-lethal effect on *An. coluzzii* is in line with previous findings [46–49] suggesting a potential additional impact of IVM on the reduction of *Anopheles* density in the field by reducing the fecundity of surviving females. The sublethal effects of IVM on *An. coluzzii* fecundity may have important implications for mosquito population dynamics and malaria transmission. In areas where repeated IVM MDAs campaigns are implemented, a decrease in the number of laid eggs could lead to a gradual reduction of adult mosquito population over successive generations. Mosquito population decreasing would reduce human-vector contact rates and consequently the entomological inoculation rate. In this study, the highest IVM concentration (C1 = 112ng/mL) did not impact the fecundity of *Ae aegypti*. This result is consistent with a previous study that showed only concentrations ≥250ng/mL are able to induce an impact on *Aedes* fecundity [17].

The minor limitation of the present study is that all experiments were performed with diluted chemical IVM mixed with blood instead of blood directly sampled on treated individuals during MDAs. Nonetheless, there are key contributions of this study to this important research topic. Firstly, it takes an integrated approach by simultaneously testing the major vectors of malaria parasites, *An. coluzzii*, and dengue viruses, *Ae. aegypti*. Secondly, this study used F1 mosquitoes derived from wild-type strains with a proven resistance status to pyrethroids. Using F1-generation mosquitoes with known age was essential to accurately isolate the specific insecticidal effect of IVM. In contrast to field collected mosquitoes, the F1 individuals reared under controlled laboratory conditions are less exposed to natural pathogens and environmental stressors, allowing for a clear assessment of IVM impact. This minimizes confusions in natural mortality and mortality induced by IVM. Finally, the concentrations tested were based on IVM plasma levels measured in humans following high-dose (3x300 µg/kg) MDA of IVM from a recent cluster-randomized trial [26].

## Conclusion

This study demonstrates that mean plasma concentrations of IVM achieved following high dose MDAs can induce high mortality in wild type *An. coluzzii* mosquitoes. In addition to mortality, this study also showed that the fecundity of surviving *An. coluzzii* females was considerably reduced even at the lowest concentration tested. However, the human IVM plasma concentrations tested did not impact either mortality or fecundity in *Ae. aegypti* mosquitoes. Therefore, standard IVM drug formulations used in MDAs may be efficacious in controlling malaria spread by *An. coluzzii* in Burkina Faso, but these MDA could not be expected to simultaneously control dengue transmission by *Ae. aegypti* in the same treated regions. Further studies combining human MDAs using different approach of IVM-based interventions, as well as livestock treatment, perhaps both with long lasting IVM formulation chemistries could be more beneficial to achieve these goals. Regardless past and current results testing IVM against some vectors, offer promise for vector-borne diseases control. Continued research and careful implementation of studies and control interventions will be crucial to maximize the benefits of IVM while minimizing potential drawbacks.

## Supporting information

S1 Fig IVM dilution and concentration preparation. (TIF)

S2 Fig *An. coluzzii* dose-response curves to IVM at days 1, 3 and 7. (TIF)

S1 File Database. (CSV)
